# Supplementary material for: Thymus atlanticus: A Source of Nutrients with Numerous Health Benefits and Important Therapeutic Potential for Age-Related Diseases
Source: Nutrients. 2023 Sep 21;15(18):4077. doi: 10.3390/nu15184077 (PMC10534698; doi:10.3390/nu15184077)
Supplement: Supplementary file 1 [file nutrients-15-04077-s001.zip › nutrients-2619339-supplementary.pdf]

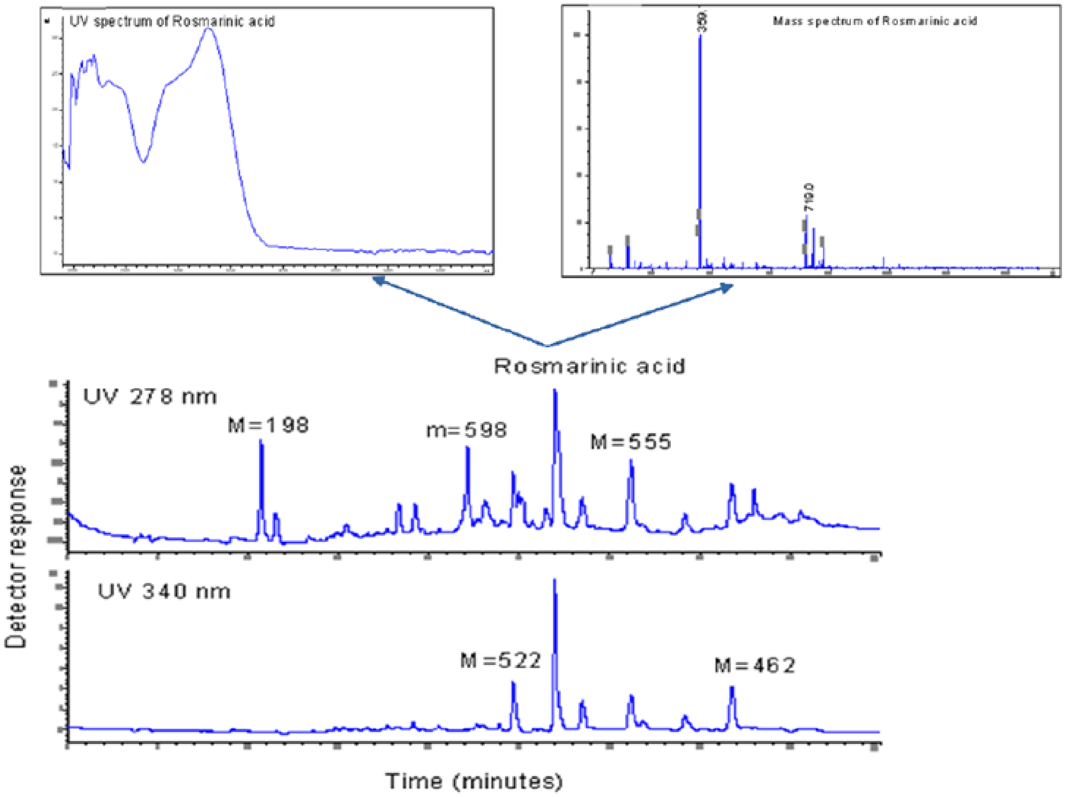

Figure S1: Chromatograms of methanolic extract of *Thymus atlanticus* obtained by RP-HPLC. At the top of the chromatogram, UV spectra (left) and mass (spectra right) (Khallouki et al., unpublished data)
